# Addressing Childhood Malnutrition in Europe: Policy Approaches to Promote Healthy Eating in Young Children

**DOI:** 10.3390/children13020213

**Published:** 2026-01-31

**Authors:** Sofjana Gushi, Olga Chouliara, Paraskevi Apeiranthiti, Dimitra Panagiotidi, Grigoris Risvas, Stavros P. Derdas

**Affiliations:** 1 Molecular Diagnostics Laboratory, KARYO Ltd., 54623 Thessaloniki, Greece; 2 Department of Dietetics, School of Health, Aegean College, 10564 Athens, Greece; 100-22388@aegeancollege.gr (O.C.); 110-12073@aegeancollege.gr (P.A.);; 3 Department of Pathology, Veterans Administration Hospital (NIMTS), 11521 Athens, Greece; 4 Genotypos S.A., 11528 Athens, Greece

**Keywords:** childhood malnutrition, obesity prevention, undernutrition, public health policy, nutrition education, Europe, WHO, EU action plan, school meals, early-life nutrition

## Abstract

**Highlights:**

**What are the main findings?**

- Europe faces a persistent double burden of childhood malnutrition, with undernutrition and obesity coexisting and disproportionately affecting vulnerable groups.
- Existing EU and WHO frameworks have improved awareness, but remain fragmented, lacking harmonized monitoring and coordinated implementation across Member States.

**What are the implications of the main findings?**

- A unified European strategy is required, integrating harmonized dietary guidelines, universal screening, fiscal measures, and school-based nutrition policies to address inequalities.
- Implementing a coordinated roadmap across short-, mid-, and long-term horizons can strengthen prevention of malnutrition and support healthier developmental outcomes for all children.

**Abstract:**

Childhood malnutrition remains a pressing public health challenge in Europe, where stunting, wasting, and underweight coexist with rising rates of childhood overweight and obesity. This policy review provides a strategic roadmap for promoting healthy nutrition in early childhood by synthesizing WHO and EU guidance and proposing coordinated action across three time horizons. Short-term goals (1–3 years) include harmonizing food-based dietary guidelines, implementing universal nutrition screening in pediatric care, and strengthening breastfeeding-supportive environments. Mid-term priorities (3–7 years) focus on fiscal levers, such as sugar taxes and healthy food subsidies; reformulating children’s products; and embedding nutrition education within school curricula. Long-term strategies (7+ years) emphasize harmonized EU-wide monitoring systems, alignment of early-life nutrition with social protection policies, and sustained investment in research on the DOHaD. Through a unified, multisectoral strategy emphasizing early-life nutrition, equitable access to healthy foods, education, and robust regulation, Europe can effectively address the double burden of malnutrition and sustainably reduce childhood obesity.

## 1. Introduction

According to the World Health Organization (WHO), childhood malnutrition is a global public health problem and refers to deficiencies, excesses, or imbalances in a person’s intake of energy and or nutrients [1]. It is a double burden to the health system, since it has two forms, undernutrition and overnutrition, raising the percentages of mortality and morbidity in both low- and middle-income countries, as well as in high-income ones [2].

The first form of childhood malnutrition, as stated, is undernutrition, manifested as stunting (low height for age), wasting (low weight for height) or underweight (low weight for age), and occurs mostly in low-income and middle-income countries [1,3]. Undernutrition also includes micronutrient deficiencies or insufficiencies, which are a lack of key vitamins and minerals, indicating nutritional inadequacy. Malnutrition throughout childhood can have major consequences regarding development and growth [3]. It is estimated that, in 2022, 149 million children under 5 years old were stunted, while 45 million were wasted [1].

Conversely, malnutrition in children may also manifest as energy overnutrition, leading to overweight and obesity. These conditions are evaluated using body mass index (BMI), derived from height and weight measurements and compared with age- and sex-specific growth reference charts [4]. Obesity refers to excessive body fat accumulation and occurs when the energy intake exceeds the energy expenditure [2]. The etiology of the problem is multifactorial and occurs from an interaction between genetic factors and the obesogenic environment and behaviors [4,5]. Childhood obesity is a pandemic, affecting nearly 37 million children below 5 years old in 2022, over 390 million children and adolescents aged 5 to 19, and affecting low- and middle-income countries as well as high-income ones [1,6]. This pandemic affects European countries as well, burdening the public health systems with rising rates of mortality and morbidity, while also compromising the quality of life of the affected children [2]. Despite efforts to reverse the rising trend by developing and implementing interventions, the problem has been exacerbated [3,7].

### 1.1. Epidemiology of Malnutrition in Europe

#### 1.1.1. Trends

The double burden of malnutrition affects European countries as well, driven by rapidly changing food environments, such as availability, accessibility, and affordability of low-cost and low-quality foods; urbanization, which is related to sedentary behaviors; and globalization [8,9]. While undernutrition and its consequences remain of concern in certain populations across the region, overnutrition, manifested as overweight and underweight, has been rising sharply across both developing and developed countries [8].

The prevalence of overweight and obesity has been increasing since the 1980s [7,10]. According to the Childhood Obesity Surveillance Initiative (COSI), which measures anthropometric data of children aged 6 to 9, the levels of overweight and obesity in many European countries have either plateaued or increased during the last decade. More specifically, the 6th COSI analysis, during 2022–2024, revealed that 25% of children aged 7 to 9 years lived with overweight, including obesity, and 10% were affected by obesity [1]. In both COSI and this review, the focused group is between the ages of 6 to 9 years old, but the tendency of overweight and obesity increases with age.

There are few studies for the prevalence of undernutrition’s consequences, like thinness and underweight, in children between the ages of 6 and 9 years old living in Europe, since most of the studies were focused on children under 5. Interestingly, even in low- and middle-income nations, where undernutrition throughout infancy and childhood is widespread, overweight and obesity rates are rising, mainly due to exposure to low-cost and low-quality meals high in fat, sugar, and energy density. Given this, the consequences of undernutrition might be less severe, but the problem remains unresolved [8].

#### 1.1.2. Differences Among Central, Southern, and Eastern Europe

Prevalence of childhood overweight and obesity varies markedly across European countries, with the overall prevalence of overweight ranging from 9% to 42%, and obesity from 3% to 20% [1]. Southern European countries, such as Cyprus, Greece, Italy, and Spain consistently report some of the highest prevalence rates of overweight/obesity, whereas Northern European countries, including Denmark, Norway, and France, demonstrate comparatively lower levels. Data from previous rounds of COSI further highlighted geographical differences, demonstrating that children in Northen Europe were the tallest, while those in Southern Europe had the highest body weight [11]. Nonetheless, there is a significant variety in each region, driven by factors such as genetics, dietary habits, socioeconomic status, and broader environmental causes [8].

Although overweight and obesity have been the primary focus of research and policy, undernutrition remains an underexplored issue in Europe. In particular, the prevalence of underweight among children and adolescents is higher in Eastern Europe compared with other regions, estimated at 8–9% [12]. This highlights the persistence of the double burden of malnutrition, where undernutrition and overnutrition coexist within the same populations, creating complex challenges for public health systems.

#### 1.1.3. Groups at High Risk

**Figure 1:**
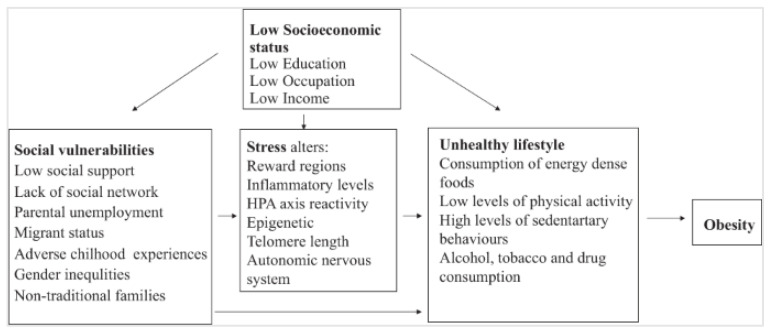
Conceptual pathway linking social vulnerabilities, lifestyle behaviors, childhood obesity, and progression to metabolic disorders, reproduced with permission from Iguacel et al., 2021 [13].

Malnutrition, whether undernutrition or overnutrition, disproportionately affects certain groups, shaped by socioeconomic, biological, and environmental factors. Children from low socioeconomic backgrounds are at particular risk for both extremes, since low socioeconomic status has been associated with limited access to food and health services, predisposing this group to undernutrition, while simultaneously increasing reliance on inexpensive, energy-dense foods that promote overweight and obesity [13]. In this context, parental education, especially maternal, is also important, because it affects health behaviors during prenatal and perinatal periods that may elevate the risk for obesity, like smoking, or for underweight and stunting [13,14]. Another population facing elevated vulnerability is migrants, due to barriers in accessing healthcare systems, language barriers, and cultural transitions. Interestingly, migrants often arrive with a healthy body weight in new countries, but the previously mentioned factors combined lead to weight gain during acculturation [15]. Adverse childhood experiences, encompassing household dysfunction, exposure to violence, and child maltreatment, has also been linked to childhood overweight and obesity [16]. Finally, hospital-related undernutrition remains a persistent yet under-recognized problem, with limited data across Europe. Even in high-income settings, such as Belgium, the condition often goes unnoticed, as fewer than one-third of acutely malnourished children receive appropriate nutritional support, leading to prolonged hospital stays and increased healthcare burden [17]. This interplay of socioeconomic, biological, and psychosocial vulnerabilities ultimately contributes to a pathway from unhealthy lifestyle behaviors and early obesity towards metabolic complications, as illustrated in Figure 1.

### 1.2. Consequences of Malnutrition

Malnutrition during childhood has particularly harmful consequences, since this period represents a crucial window for development, considering that the phenotype is affected by nutritional influences. As mentioned above, undernutrition includes stunting, wasting, or underweight. These manifestations are life-threatening, due to increased susceptibility to infectious diseases, and can lead to irreversible long-lasting consequences, including impaired physical growth and cognitive development, and reduced sensory-motor abilities [3,18]. Delayed child neurodevelopment impairs intellectual capacity and educational accomplishment and, in the long run, severely influences the human output in adulthood and the economic development [3]. Furthermore, underweight is related to lower BMI and fat-free mass, and higher fat mass in adults, while, in cases of rapid BMI gain, there are also adverse effects, such as increased risk for central adiposity, as well as noncommunicable diseases [19,20]. Undernutrition also includes deficiencies or insufficiencies of micronutrients crucial for growth, immunity, and brain development, such as iron deficiency causing anemia [21].

Obesity can also influence growth and development in puberty, causing acceleration of linear growth velocity, advanced bone age, and, often, early puberty, due to hormonal imbalances [22]. Regarding children living with overweight, including obesity, over 60% of them will be overweight in early adulthood, depicting the major health crisis that needs to be addressed [7]. These growing rates imply higher prevalence of obesity’s comorbidities in much younger populations, such as higher blood pressure, higher blood cholesterol, and insulin resistance, increasing the risk of cardiovascular diseases [2]. Childhood obesity predisposes individuals to noncommunicable diseases, including type 2 diabetes, osteoarthritis, some types of cancer, and, as mentioned before, cardiovascular diseases, which are responsible for 77% of the burden of disease and almost 86% of premature mortality [7,23]. Furthermore, obesity impacts adversely neurodegenerative and autoimmune disorders, such as rheumatoid arthritis, inflammatory bowel syndrome, Hashimoto’s disease and systemic lupus erythematosus, as well as prostate and respiratory diseases, like asthma and obstructive sleep apnea [2,23]. Another common problem among individuals with overweight and obesity is musculoskeletal and orthopedic problems [9].

It is very common that children with overweight and obesity experience weight stigma, in the forms of victimization, teasing, and bullying in school settings and in home settings. Discrimination against obese patients also takes place in healthcare and media. All of these contribute to increased sensitivity to depression, low-self-esteem, and negative body image, leading to social isolation and poor academic outcomes, limited physical activity, and disordered eating patterns [24]. There are associations between weight-based discrimination and increased body weight in youngsters, making it urgent to advocate against weight stigma. Since obesity reduces quality of life and leads to worse physical well-being, patients are susceptible to mental illnesses, including mood, anxiety, and personality disorders [2,22,25].

### 1.3. Biological Bases

#### 1.3.1. Maternal Factors

A multitude of studies have shown that malnutrition is creating an intergenerational cycle. More specifically, stunted women face the risk of delivering an infant of low birthweight [1]. Respectively, a higher pre-pregnancy BMI is associated with childhood overweight and its effects, such as higher glucose levels, waist circumference, adiposity, and insulin resistance [26]. The risk for obesity and symptoms of metabolic syndrome during childhood is exacerbated by gestational diabetes, which alters the infant’s body composition, leading to increased fat mass and, in the case of females, greater odds that their offspring will also live with overweight and obesity [27]. In most of the cases, gestational diabetes is a consequence of obesity, highlighting the necessity for interventions to promote healthy nutrition and physical activity among girls during adolescence and young adulthood, or to prevent excess weight gain during pregnancy [28]. Furthermore, many studies have shown that excess weight gain during pregnancy is also associated with childhood overweight, higher systolic blood pressure, and C-Reactive Protein (CRP), among others [26]. However, both under- and overnutrition during pregnancy may affect the risk of obesity and disease during adulthood.

#### 1.3.2. Epigenetics

In recent years, increasing attention has been directed towards the role of epigenetic mechanisms in affecting health outcomes. Epigenetics refers to heritable changes in gene expression that occur without alterations in the underlying DNA sequence, such as DNA methylation, histone modification, and non-coding RNA regulation, leading to on- or off-regulation of critical genes [22,29]. These mechanisms are highly responsive to environmental cues, with nutrition being one of the most crucial modulators [26,30].

The first 1000 days of life, from fertilization to 24 months of a child’s life, are a critical window of developmental plasticity, during which the genome is very sensitive to environmental exposures, determining health and risk of diseases, as supported by the Developmental Origins of Health and Disease (DOHaD) concept [31,32]. For instance, adverse nutritional conditions during the prenatal period, such as undernutrition, affect postnatal health and growth and increase the risk of chronic diseases [30]. Conversely, diets enriched with nutrients acting like methyl donors, like folate, methionine, choline, and vitamins B6 and B12, during pregnancy are linked to reduced risk for disease, enhanced brain development, and even protection against certain cancers [29]. Beyond single micronutrients, broader dietary patterns have been shown to influence epigenetic programming. Following a Western diet during pregnancy, which is high in saturated fats and sugars, but deficient in fruits, vegetables, whole grains, and seafood, may result in physiological dysfunctions. In contrast, following a Mediterranean diet in early pregnancy, characterized by an abundance of fruits, legumes, whole grains, monounsaturated and polyunsaturated fats, is associated with positive neurobehavioral outcomes [30].

Epigenetic alterations are additionally influenced by environmental chemicals that disturb the endocrine system, and exposure to these, especially in early life, is linked to delayed development, diseases, and some types of cancer [30]. Similarly, maternal smoking and alcohol consumption during pregnancy have been shown to induce adverse epigenetic changes with lasting consequences for growth, neurodevelopment, metabolism, and overall health [30,33]. Finally, psychosocial and environmental stressors, as well as adverse childhood experiences, elevate the risk for noncommunicable diseases by affecting the epigenome [34].

It is now widely recognized that the interplay between genes and the environment affects gene expression through epigenetic mechanisms, with both beneficial and harmful impacts on health [22]. These epigenetic changes, inherited from generation to generation, contribute to the intergenerational cycle of malnutrition. Breaking this cycle requires identifying critical windows of plasticity and knowing how specific nutrients and metabolic conditions shape the epigenome. Such knowledge can pave the way for targeted, personalized nutritional interventions designed not only to prevent, but potentially to reverse, adverse health outcomes [29].

### 1.4. The Importance of Dietary and Lifestyle Interventions During the First 1000 Days

The first 1000 days of life represent the period from the conception to the second birthday of the newborn. This is a period of rapid growth and maturation of the endocrine, neural, and metabolic pathways, affected by exposures both in utero and in the postnatal environment [35]. During this period, it was noted that there are three crucial stages that may affect survival, susceptibility to diseases and infections, or growth and development throughout the course of life [26].

The first critical stage is the prenatal period, in which maternal nutritional exposures influence the growth patterns and the metabolism of the offspring [26]. Furthermore, during the prenatal period, insufficient maternal dietary intake affects the development of the fetus and the risk of childhood underweight, stunting, and deficiencies in micronutrients [19]. Smoking during this period can also contribute to a lower birthweight and reduced anthropometric measurements in general [33]. Overnutrition, on the other hand, leading to pre-pregnancy overweight or excessive weight gain, is linked to childhood obesity, due to increased exposure to nutrients through the placenta. In addition, genetic predisposition to elevated maternal BMI correlates with higher birth weight in newborns [26].

The second crucial stage is the postnatal period, 6 to 24 months, and the nutritional exposures of the infant during this. According to guidelines, exclusive breastfeeding is recommended for the first six months, since it is beneficial for both the mother and the infant, as it has been confirmed [36]. Exclusive breastfeeding has been associated with healthy gut microbiome and immune system; decreased risk of gastrointestinal infections, asthma, and other pathologies such as celiac disease and inflammatory bowel diseases; and, lastly, with good neurobehavioral development [37,38]. Regarding obesity, exclusive breastfeeding is thought to have a protective role against it. Breast-fed infants present a slower growth curve, likely due to lower plasma Insulin-like Growth Factor-1 (IGF-1) [39]. This might be attributed to the fact that breast milk is lower in energy and protein density and higher in fat, compared to formula [26,39]. Another possible explanation for the protective role of breastfeeding is that breastfed children tend to be more developed in self-regulation [26].

There are plenty of nutritional exposures from 6 to 24 months that play pivotal roles in determining the risk for childhood obesity. The first risk factor is rapid weight gain during these months, which is measured by growth charts [5]. Another factor that has received a lot of attention is the timing of solid foods. Particularly, introducing complementary foods before the age of 4 months, especially to formula-fed children, was linked to increased odds for obesity [40]. A higher BMI in children later in life, and increased adiposity, was also linked to high dietary protein intake, especially from animal sources, highlighting the important role of dietary intake during crucial developmental stages [41,42]. Controlling parental feeding practices or not responding to indications of hunger and satiety have been associated to increased BMI and risk for overweight and obesity, similar to adverse childhood experiences [43].

During postnatal maturation, the microbial colonization of the infant’s gastrointestinal tract occurs, affecting metabolic, immune, and neural processes. The development of an infant’s microbiome is shaped by both milk and introduction of solid food, but also by illness, antibiotic exposures, and other environmental factors [26,35]. Damage to the microbiome composition, known as dysbiosis, has been associated with obesity, type 1 diabetes, inflammatory bowel disease, celiac disease, neurodevelopmental disorders, allergies, and asthma [35,44].

The period from conception to 24 months is characterized by high sensitivity and vulnerability to maternal and environmental exposures. The timing and quality of complementary food introduction are critical determinants of infant nutrition and long-term health. Introduction of solid foods around 6 months of age is recommended, as earlier introduction is associated with increased risks of overweight and infections, whereas delayed introduction may contribute to micronutrient deficiencies and feeding difficulties. Moreover, early exposure to energy-dense, sugar- and salt-rich foods, and non-responsive feeding practices, may promote excessive energy intake and increase the risk of later overweight and obesity. Providing optimal early-life nutrition lays the foundation for healthy development and long-term health outcomes. Consequently, this period is increasingly regarded as a vital window of opportunity for future preventive interventions.

## 2. Strategic Framework at the EU and WHO Levels

The European Union (EU) and the WHO have both developed strategies to address childhood obesity and malnutrition throughout Europe. These frameworks aimed to tackle the increased prevalence of obesity, improve children’s nutrition environments, and promote healthy eating habits.

### 2.1. EU Action Plan on Childhood Obesity (2014–2020)

The EU Action Plan on Childhood Obesity was launched in 2014 with the primary goal of combating the growth in obesity among children (0–18 years old) by 2020. This program outlined several key areas to tackle childhood obesity in a comprehensive manner. First, it emphasized the importance of supporting a healthy start in life, including the mother’s pre-conception weight, her weight gain during pregnancy, breastfeeding, and the early-life nutrition of the baby. In addition, it sought to promote healthier school environments by ensuring access to nutritious school meals, with special focus on the socially disadvantaged children, while also transforming schools into spaces that encourage regular physical activity. Another important pillar was to make better food options both affordable and appealing, complemented by clear product labelling and consumer information to assist families in selecting nutritional options. At the same time, the program highlighted the need to limit the marketing and advertising of foods high in fat, sugar, and salt to children, as there is a clear correlation between the prevalence of overweight and obesity in children and screen/TV exposure. Moreover, it encouraged informing and educating families to cultivate healthier family meals and pay closer attention to children’s diet. Alongside nutrition, the promotion of physical activity was strongly recommended as an essential preventive measure. Finally, it underlined the necessity of strengthening monitoring and evaluation of children’s health stats, including diet and physical activity, while also improving the systematic data collection of the research [45].

Evaluations highlighted both progress and challenges. The mid-term evaluation in 2018 found that all participating countries were active in more than one of the areas of action. In general, the Action Plan proved beneficial, serving as a guide tool, providing awareness, motivation, examples, and guidance. The areas of actions that were more successful were the promotion of healthier school environments through nutritious school meals, the creation of physical activity-friendly settings, and the encouragement of regular physical activity, alongside systematic data collection. On the other hand, the actions aiming to make healthier food options more affordable and appealing through clear labelling and consumer information, as well as those intended to limit the marketing and advertising of foods high in fat, sugar, and salt to children, require additional action and support [46]. SAFE’s 2020 assessment pointed out that the Action Plan’s goal of combating the rise of childhood obesity in Europe had not yet been fully achieved because the rate of childhood obesity continued to rise. The Action Plan faced strong challenges in limiting the marketing and advertising of unhealthy foods to children and in promoting and sustaining physical activity among young populations, but it also helped to provide a structured framework to support Member States’ efforts and facilitate the development of specific interventions [47].

The EPHA’s 2021 debate emphasized that the ultimate goal of halting the epidemic of obesity was still not within reach, with nearly 30% of European children overweight and obese. To address this major problem, strong political leadership was considered essential, requiring the European Commission to align national activities. The debate also pointed to Europe’s Beating Cancer Plan as the main opportunity for follow up to the 2014–2020 Action Plan. Finally, the discussion underlined the relevance of the growing evidence base, supported by projects such as the Horizon 2020 STOP project and WHO data, in guiding future policies and interventions [48].

### 2.2. WHO European Food and Nutrition Action Plan (2015–2020)

In parallel, the WHO European Food and Nutrition Action Plan (2015–2020) encouraged a Whole-of-Government, Health in All Policies approach to reduce the burden of avoidable diet-related noncommunicable diseases (NCDs), obesity, and malnutrition. Its main objectives were the creation of healthier food and drink environments, the promotion of healthy diets, especially for vulnerable groups, and the reinforcement of health systems to better support nutrition. In addition, the plan emphasized the importance of stronger surveillance, monitoring, evaluation, and research, as well as the need for improved governance to ensure a Health in All Policies approach [49]. Nutrition education in kindergarten should not be treated as a short-term intervention, as eating behaviors and food preferences are shaped gradually during early childhood. Short, isolated programs tend to produce transient effects, particularly when not reinforced by the home and food environment. Sustainable dietary change requires long-term, repeated, and developmentally appropriate education integrated across school and family settings.

**Figure 2:**
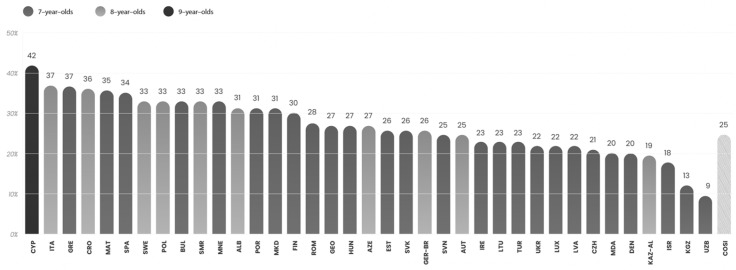
Prevalence of overweight (including obesity) in children ages 6–7 years across 37 countries, based on WHO European Childhood Surveillance Initiative (COSI), Round 6 (2022–2024). Source: World Health Organization Regional Office for Europe; COSI Round 6 (2022–2024). License: CC BY-NC-SA 3.0 IGO [1]. Note: The figure shows that, although the average prevalence of overweight among children aged 6–7 years is around 25% in the WHO European Region, the rates vary widely among countries, highlighting significant regional disparities.

A key monitoring tool was the WHO European Childhood Obesity Surveillance Initiative (COSI), which collected data on children aged 6–9 years old across Europe [50]. The latest COSI (Round 6), was conducted between 2022 and 2024 and measured around 470,000 children in 37 different countries in the region, reporting that around 25% of children were overweight (including obesity). However, the rates ranged significantly, from 9% in some Northen countries to over 42% in parts of Southern Europe. This variation is illustrated in Figure 2, which highlights the marked disparities among countries, with some showing stabilization, while others continued to face rising rates [1]. Taken together, both the plans (EU and WHO) were ambitious and provided valuable roadmaps for tackling childhood obesity. However, their overall impacts were limited because of lack of harmonization and insufficient evaluation.

### 2.3. A Comparative Overview of National Programs

National programs across Europe vary widely, and they show different policy priorities. The main areas of intervention across the region include school meal provisions, taxation of sugar-sweetened beverages (SBB) and front-of-pack labelling schemes. Table 1 provides a comparative overview of these measures across selected countries.

School meal programs are very different across Europe. For example, some countries, such as Finland and Sweden, provide free school meals to all children [51,52]. In contrast, in Denmark, meals are usually paid by parents or provided by municipalities [53]. In Greece, the ‘School meals’ program delivers daily hot meals in public schools, and it targets disadvantaged populations, which highlights the role of social policies in promoting equality [54]. In Ireland, the School Meals Scheme has recently extended, addressing nutrition, as well as social equity [55]; meanwhile, in Luxembourg, the main goal was to make school meals sustainable, with an emphasis on local, organic ingredients [56]. Other examples include the Dutch ‘Schoolmaaltijden’ Program, which provides meals to children at risk of food insecurity [57], and the Polish ‘School Meals Program/Meals at School and at Home’, which combines education with support for low-income families [58]. Last, Austria joins with a focus on providing fruits, vegetables, and milk in schools [59]. These instances demonstrate discrepancies in access across Europe.

Some governments apply tariffs on sugar-sweetened beverages (SBB) to make consumers buy less of them and corporations reformulate those products [60]. France introduced the taxation in 2012 and updated it in 2018. Nutri-Score is a front-of-pack labeling system that rates the nutritional quality of foods on a color-coded A–E scale based on an algorithm balancing unfavorable nutrients (energy, sugars, saturated fats, sodium) against favorable components (fiber, protein, and fruit, vegetable, legume, nut, and selected oil contents). Although intended to support healthier food choices, Nutri-Score assesses foods per 100 g, without considering portion size, food matrix, degree of processing, or overall dietary context. Consequently, some ultra-processed or reformulated products may achieve favorable scores, while minimally processed, nutrient-dense foods may be penalized due to their fat or energy content, leading to potential misclassification. These limitations are particularly relevant in early-life nutrition, where age-specific nutritional requirements are not captured by simplified nutrient profiling systems. This led companies to lower sugar levels [61,62]. Portugal started similar tax in 2017, with some positive results [63]. Ireland followed in 2018, reporting lower sugar consumption [62]. Hungary also adopted a public health product tax with evidence of measurable impact, as shown in recent evaluations [64]. On the other hand, Italy delayed and planned it for 2026 [62]. These examples indicate that SSB taxes can have a positive influence, although not all governments have chosen to impose them.

The final intervention was front-of-pack labels, which helped many individuals choose healthier products. Some countries, like Belgium, Switzerland, Germany, France, Spain, Luxembourg, and the Netherlands, have adopted the Nutri-score [65,66], while the Nordic countries use the Keyhole logo [67,68,69]. Finland uses the Heart symbol [70]. However, on the European level, there is still no mandatory label, and all countries use different systems, indicating a lack of uniformity. To summarize, several European countries have begun key initiatives, yet there are still significant gaps and differences among countries. A comparative overview of these measures is presented in Table 1.

**Table 1:** Comparative overview of national measures addressing childhood obesity in selected European countries (2025).

Country	SSB Tax (Year/Structure)	FOP (Nutri-Score Status)	School Meals (Program)	Refs.
Greece	-	Nutri-Score (voluntary)	Targeted hot meals in primary schools (231,062 meals/day, 2024/2025), mainly for disadvantaged kids	[54]
Ireland	2018	Nutri-Score (voluntary)	Expanded Hot School Meals Scheme (2025); focus on nutrition and social equality	[55,62]
France	2012	Nutri-Score (2017)	Free in some municipalities; in most areas, families pay depending on household income	[51,60,61,62,66,69]
Portugal	2017	-	Free for children from low-income families or with disabilities	[60,62,63]
Spain	2017	Nutri-Score (2021)	Free for children from low-income families and for disadvantaged kids	[62,65,66,69]
Italy	Expected 2026	NutrInformBattery	No national school meal program information	[62]
Germany	-	Nutri-Score (2020)	Free meals for children from low-income households; others pay a contribution	[65,66,69]
Belgium	2015	Nutri-Score (2019)	Limited information available	[62,65,66,69]
Netherlands	2024	Nutri-Score (2021)	Meals in 2379 schools; program aims to reduce child hunger by 30%	[57,60,61,62,66,69]
Luxembourg	-	Nutri-Score (2021)	Free for children from low- income families or socially excluded families	[56,65,66,69]
Austria	-	-	EU School Scheme (fruits, vegetables, milk)	[59,66]
Finland	2011	Heart symbol	Universal free school meals for all students (850,000 children all educational levels)	[51,52,60,70]
Sweden	rejected	Keyhole	Universal free school lunches	[51,52,67,68,69]
Denmark	rejected	Keyhole	No universal scheme; meals usually paid by parents or municipalities	[53,67,68,69]
Poland	2021	Keyhole	‘Meals at School and at home’ free for children from disadvantaged families	[58]
Hungary	2011	-	Free for disadvantaged kids	[60,64]
Slovakia	2025	-	Free for children from low-income households and for the last year of preschool	[62]

### 2.4. Strengths and Gaps

A review of the European strategies, as well as national programs, reveals certain achievements, but also major gaps that restrict their overall effectiveness in addressing childhood obesity and malnutrition.

To begin with the strengths, various measures have proven beneficial, particularly those involving front-of-pack labelling, free school meals, and breastfeeding advocacy. One of the most effective tactics in Europe is the promotion of healthier food options through labelling programs. Several countries have implemented a front-of-pack labelling system. For example, Finland applied the Heart symbol [71]; Sweden, Norway, Denmark, Iceland, Lithuania, and North Macedonia use the Keyhole logo, which is based on Nordic Nutrition recommendations [72,73,74]; and countries like France, Belgium, Switzerland, Germany, Spain, Luxembourg, and the Netherlands adopted the Nutri-Score [74,75]. Another important aspect is that some European countries provide free school meals. For instance, Finland and Sweden provide universal free school meals to all students, ensuring that every child has the opportunity to grow and develop healthily [76,77]. Lastly, the promotion and preservation of breastfeeding have received priority. The WHO European Food and Nutrition Action Plan 2015–2020 highlights the value of promoting a balanced diet prior to conception, during pregnancy, and for infants and children in order to maintain healthy growth and development to prevent childhood obesity and other NCDs. As a result, Member States were urged to implement the International Code of Marketing of Breastmilk Substitutes and to strengthen the baby friendly hospital initiative, the capacity of health providers and services to support optimal child feeding through appropriate training, good maternity care practices, and early childhood services to support breastfeeding [78]. Also, Regulation (EU) 2016/127 provided specific instructions for the composition and labelling rules for the infant formula market [79].

Despite these qualities, there are considerable gaps that limit the effectiveness of the existing tactics. First, there is an obvious paucity of monitoring data. Programs such as WHO’s COSI gather useful information, though the data is not always consistent among nations and there is no single EU-wide system. These facts make it hard to compare results [80] and is also reflected in the European Court of Auditors special report 23/2024, which concludes that food labelling practices remain fragmented across Europe and consumers “can get lost in the maze of labels” [81].

Policy implementation varies greatly among countries. Some countries, for example, provide free school lunches, or they apply sugar taxes and require food labelling, while others do far less. This creates unequal conditions for children across Europe [82]. A comprehensive EU-level assessment by Guio et al. (2023) [74] confirmed these disparities, showing that only a few Member States, such as Finland, Sweden, Estonia, Latvia, and Lithuania, offer universal free school meals, while most others rely on targeted schemes that reach only a fraction of children in need. This study also estimated that achieving the Child Guarantee objective of ensuring one healthy free school meal per school day for all children at risk of poverty would require less than 2% of annual education spending in most countries, an affordable and socially beneficial goal that could significantly reduce inequalities in access to adequate nutrition.

Furthermore, according to the findings of the 6th Round of WHO’s COSI (2022–2024), there were significant discrepancies in the ranges of childhood overweight in several northern countries vs. some southern regions of Europe [83].

Third, the evaluation of policy is frequently inadequate. The mid-term evaluation of the EU Action Plan on Childhood Obesity (2018) found that, while Member States were engaged in numerous areas, progress was inconsistent, and many pledges needed follow up [2]. Similarly, the European Court of Auditors (2024) highlighted that enforcement of food labelling was varied across countries, and monitoring mechanisms were insufficient, leaving consumers with limited protection [73].

## 3. Roadmap of Europe

**Figure 3:**
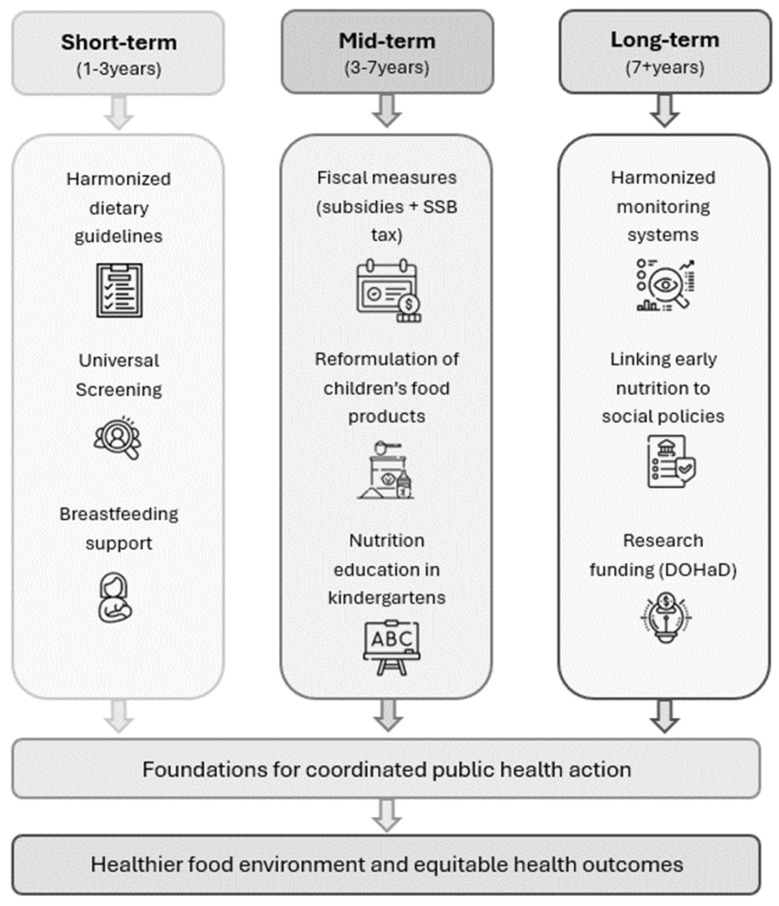
Roadmap of Europe for Preventing Childhood Undernutrition and Obesity. A brief summary of the proposed roadmap. Made by Paraskevi Apeiranthiti.

The following section proposes a plan to increase the prevention of childhood obesity and undernutrition in Europe, building on existing EU and WHO policies. Although existing initiatives have resulted in significant success, several problems persist, including country differences and poor action coordination. A unified, step-by-step approach is, therefore, required to ensure more effective and equitable implementation across the region. The roadmap described here is divided into three time horizons, short-term (1–3 years), mid-term (3–7 years) and long-term (7+ years), ensuring both immediate action and sustainable impact. Each phase focuses on specific actions that aim to create healthier food environments, strengthening public health systems and promoting better nutrition for all children in Europe. The proposed roadmap is summarized and depicted in Figure 3.

### 3.1. Short-Term Actions (1–3 Years)

In the short term, efforts should be directed toward practical interventions that can be rapidly implemented within existing health, education, and social systems. These policies seek to make apparent progress in early childhood nutrition and obesity prevention, while promoting better parity among European countries. The proposed goals include harmonizing nutritional requirements for children, developing universal screening tools for malnutrition and overweight during pediatric check-ups, and encouraging breastfeeding through enhanced workplace and community support. Taken together, these attempts can lay the groundwork for a more coordinated and preventive public health approach across Europe.

#### 3.1.1. Harmonization of Dietary Guidelines

The alignment of dietary standards across Europe constitutes a critical first step towards a more coordinated approach to child nutrition. At the moment, each European country has its own food-based dietary guidelines (FBDGs), which are useful, but frequently vary from country to country. To make them more comparable, the European Food and Safety Authority (EFSA) and the Joint Research Center (JRC) might collaborate to develop a single reference framework for children’s nutrition [75,76]. This framework would not replace national rules, but it would help countries utilize similar terminology, portion sizes, and nutrient targets. It should also adhere to the WHO nutrient profile model and the European Commission’s guidance on Food-Based Dietary Guidelines [77,78]. These guidelines often address the main food and nutrient groups, including starchy foods, fruits and vegetables, milk and dairy products, legumes, meat, fish and oils, sweets and snacks, water, non-alcoholic beverages, salt, and sugars. Using a similar strategy allows for easy comparison and tracking of progress across countries. Long-term, harmonized dietary guidelines could support more effective public health policies, foster collaboration among Member States, and assist in eliminating inequities in children’s diets throughout Europe.

#### 3.1.2. Universal Malnutrition/Overweight Screening at Pediatric Check-Ups

Moving on, regular screening for malnutrition and overweight is a crucial step for early detection and intervention. Many European countries lack a standardized approach, making it difficult to identify at-risk children and compare data across regions. The introduction of a universal screening method during routine pediatric check-ups could benefit health professionals in detecting undernutrition and excess weight at an early stage [79]. There are several validated screening tools available, such as the Malnutrition Universal Screening Tool (MUST) developed by the British Association for Parental and Eternal Nutrition [81] and the Screening Tool for the Assessment of Malnutrition in Pediatrics (STAMP) which is used in hospitalized children aged 2 weeks to 16 years [80,82]. These tools are straightforward, useful, practical, evidence-based, and easy to incorporate into electronic medical health records [50] or current national monitoring systems. Using such methods would improve data dependability, allow for cross-country comparisons, and boost observation networks like the COSI. In the long run, universal screening would really benefit targeted interventions, support clinical decision making, and reduce health inequalities among European children.

#### 3.1.3. Promotion of Breastfeeding

Breastfeeding support is one of the most effective early intervention strategies for improving child health and preventing obesity [82]. However, many mothers struggle to continue nursing after returning to work due to lack of facilities, restricted time off, and lack of company support [79]. Creating breastfeeding-friendly workplaces is, thus, an important short-term step toward boosting mother-and-child well-being. Evidence indicates that policies providing paid maternity leave, flexible scheduling, and private spaces for expressing milk are significantly vital to supporting breastfeeding duration and exclusivity [79,81]. Employers can also play a major role by adopting UNICEF’s recommendations on breastfeeding rooms, supportive management practices, and awareness training for staff [84,85]. Implementing these measures helps reduce stress for working women and ensures that breastfeeding remains a viable option, even in challenging work environments.

### 3.2. Mid-Term Actions (3–7 Years)

In the mid-term phase, activities should go beyond short-term behavioral improvements to address the broader economic and environmental issues that influence dietary choices. Evidence from several countries suggests that sugar taxation alone does not consistently reduce sugar consumption. In Brazil, fiscal measures targeting sugar-sweetened beverages have shown limited impact, likely due to continued affordability, substitution with other sugary products, and insufficient nutrition literacy. These findings indicate that sugar taxes should be embedded within broader strategies, including nutrition education, food reformulation, and supportive food environment policies, to achieve sustained benefits in children. These include fiscal policies, such as subsidies for healthy foods and taxes on sugar-sweetened beverages; reformulating children’s food products to reduce sugar, salt, and saturated fat; and incorporating nutrition education into the formal school curriculum beginning in kindergarten. Together, these policies can make healthy choices easier and more accessible, encourage industry responsibility, and promote long-term improvements in diet quality across Europe.

#### 3.2.1. Fiscal Measures

Fiscal policies are an affordable strategy to promote healthier eating habits and improve population nutrition. Evidence suggests that subsidies for fruits, vegetables, and other healthy foods can boost consumption, especially among low-income groups [83,86]. According to the World Health Organization and the World Bank, well-designed food subsidies can reduce retail prices, support equitable access to nutritious foods, and drive producer reformulation [84,86]. For example, the European Union’s School Scheme provides free or subsidized fruits, vegetables, and milk to schoolchildren in all Member States, demonstrating how fiscal support can effectively combine health and education objectives [87,88].

At the same time, sugar-sweetened beverage (SSB) taxes have proven helpful in lowering sugar consumption and driving product reformulation. Several European nations, including Belgium, France, Finland, Hungary, Latvia, Monaco, Norway, Ireland, and Portugal, have reported significant drops in SSB use after the tax was implemented [6]. The WHO recommends that such taxes should be significant enough, often raising retail prices by at least 20%, to change purchasing behavior and reduce obesity rates [89].

Overall, combining subsidies for healthy foods with taxes on unhealthy products represents a balanced budgetary approach that encourages positive choices and discourages negative ones.

#### 3.2.2. Reformulation of Children’s Food Products

Reformulating food products for children, which involves changing the content of a food to improve its nutritional profile, is an important mid-term measure to improve diet quality across Europe. Many products marketed to children are heavy in sugar, saturated fat (SFA), and salt, which contribute to unhealthy eating habits and increased risk of obesity. Reformulation policies are intended to encourage food manufacturers to reduce these components while maintaining product safety, quality, and flavor. According to the WHO, product reformulation regulations benefit not only public health, but also individuals and businesses. From a public health standpoint, reducing the levels of sugar, salt, and SFA levels in foods reduces the risk of diet-related NCDs and benefits all socioeconomic groups. Individuals benefit from reformulation because it improves the nutritional value of foods and promotes healthy eating habits. For businesses, it creates a fair competitive environment, offering opportunities to improve brand image while avoiding taxation or marketing restrictions on unhealthy items [90]. At the European level, the Best-ReMaP project has mapped national reformulation policies and monitoring systems across Member States, showing that most countries have already established voluntary or mandatory targets for decreasing salt, sugar, and SFA levels in processed foods [62]. Over time, the reformulation of foods that are consumed by children can make healthier options the norm, helping all families regardless of income or education to access better nutrition and supporting the long-term prevention of NCD including obesity.

#### 3.2.3. Introduction of Nutrition Education at the Kindergarten Level

Integrating nutrition education into the early years of school is an essential mid-term step toward developing healthy behaviors. The preschool and early primary years play a critical role in shaping children’s eating preferences and behaviors. Kindergartens, therefore, provide a unique opportunity to combine learning and practice through lessons, nutritious meals, and supportive and friendly environments. A good diet during childhood is key to reducing the risk of nutrition-related disorders, including obesity [91,92]. According to the WHO Food and Nutrition Policy for schools, schools are ideal settings to promote healthy eating because they may connect school meals with community involvement. It also claims that proper eating can improve children’s well-being, learning ability, academic performance, behavior, mental and social well-being, and physical well-being [93]. In addition, a systematic review, that had the main objective to research the effectiveness of dietary intervention programs in children aged 3–12 years old in schools and covered 19 studies, showed significant improvement in knowledge and healthy eating behaviors (including increased fruit and vegetable consumption and decreased consumption of sugary snacks and drinks), while several studies showed a reduction in BMI and/or weight improvement, especially when the interventions were both educational and practical, and of sufficient duration [94]. Nutrition education, thus, can contribute to long-term changes in dietary behavior and help eliminate child health inequalities across the region.

### 3.3. Long-Term Actions (7+ Years)

Long-term measures should focus on building sustainable systems that assure lasting improvements in children’s nutrition and health throughout Europe. These approaches aim to move beyond individual interventions and create a framework that combines data, social protection, and research. Over time, constant monitoring, broader social policies, and ongoing scientific investment can assist in maintaining progress, reducing inequalities, and adapting tactics to new challenges.

#### 3.3.1. Harmonized Monitoring Systems

Developing harmonized monitoring systems is a long-term priority for ensuring consistent and comparable data on children’s nutrition and obesity across Europe. Consistent surveillance enables countries to track trends, assess the results of health programs, and design better public health policies. In response to the need for standardized surveillance data on the prevalence of overweight and obesity, the WHO Regional Office for Europe established the WHO European Childhood Obesity Surveillance Initiative (COSI), which already collects valuable information for more than 40 countries and millions of children aged 6 to 9 years [95].

Recent syntheses highlight the extent and endurance of the problem: In the WHO European Region, one child out of three is overweight or obese. Over 60% of children who are overweight before puberty will be overweight in early adulthood. Children and adolescents aged 5–19 have shown rising obesity rates in almost all nations [96]. Without comprehensive and comparable monitoring, governments risk missing the true impacts of prevention programs, reinforcing the need for harmonized indicators.

However, data collection methods vary among countries, making the intercountry comparisons difficult. Recent European projects, such as DEPIPAC and the PEN consortium, have advocated for a conceptual framework to work as a comparable key for measuring comparable health indicators, hence facilitating health reporting and monitoring across Europe [97,98].

#### 3.3.2. Linking Early Nutrition to Social Policies

Linking early nutrition to social policies is an important long-term action for reducing inequality in children across Europe. The first 1000 days, from conception to age two, shape growth, brain development, and future disease risk, and socioeconomic conditions can strongly influence that window, what families are able to feed, and how they take care of infants [98,99]. The European Child Guarantee (2021) calls on Member States to ensure that children in need have access to early childhood education, healthcare, housing, and at least one healthy school lunch per day, therefore aligning nutrition to broader social security [97]. In addition, evidence from the European Commission shows that improving maternal and infant nutrition, in the window of the first 1000 days, can prevent obesity and narrow the gaps between socioeconomic groups [46]. To tackle both poverty and malnutrition, UNICEF proposes building synergies (cooperative interaction between separate entities that creates an enhanced combined effect) between child nutrition and social protection, like food support, parental leave, and family benefits [100,101,102]. Investing early in child nutrition and social safety guarantees that all children have a fair and healthy start in life.

#### 3.3.3. Research Funding

Europe continues to make significant investments in research on the Developmental Origins of Health and Disease (DOHaD), recognizing the long-term effects of early environments, nutrition, and epigenetic mechanisms on lifelong health [103]. Researchers can now study early-life stressors, nutrition, and epigenetic markers, such as DNA methylation, in a harmonized and privacy-protected manner, thanks to initiatives such as the Horizon 2020 Life Cycle Project, which established the EU Child Cohort Network that linked data from over 250,000 children and parents across Europe [104]. Complementary efforts, such as the Early Nutrition Project, have offered evidence-based guidelines for maternity, lactation, and early childhood nutrition, emphasizing their crucial roles in preventing obesity and NCDs [105]. Building upon these accomplishments, the Horizon Europe 2025–2027 Health Cluster prioritizes funding for research on environmental and social determinants of health; noncommunicable and neurodevelopmental diseases; and the use of AI, biotechnology, and FAIR data infrastructures to advance understanding of early biological programming [106].

Together, these initiatives demonstrate a clear European approach that connects early nutrition and epigenetic research with policy formulation and prevention strategies, ensuring that investment in early-life research continues to shape healthier generations in the future.

## 4. Methodology

The current research is a narrative policy evaluation based on a thorough search of European and international frameworks for combating childhood malnutrition. Relevant materials and technical reports were acquired from institutional databases such as the World Health Organization (WHO), the European Commission, the European Public Health Alliance (EPHA), and national health ministries. The examination included policy texts, surveillance data (particularly from the WHO European Childhood Obesity Surveillance Initiative—COSI), and assessments of the EU Action Plan on Childhood Obesity (2014–2020) and the WHO European Food and Nutrition Action Plan (2015–2020). Publications from 2014 to 2025 were selected to reflect current advances and policy reviews. Data were thematically evaluated to identify strengths, gaps, and emerging trends, which helped shape the recommended roadmap for future European policies.

## 5. Models of Comprehensive Child Nutrition Policy

### 5.1. Finland’s Child Nutrition Policy

Finland is one of the most successful examples of integrated child nutrition programs in Europe, with its national school lunch system being implemented universally in all public schools since the 1940s. According to the Food and Agriculture Organization (FAO), the program provides free, comprehensive, and balanced meals to all students aged 6 to 16, with an emphasis on nutritional adequacy, sustainability, and healthy eating instruction [107].

In accordance with studies, school meals in Finland contribute significantly to children’s overall dietary quality, covering up to one-third of their daily energy demands and lowering unhealthy food consumption outside of school [108].

In parallel, the “Tasty School” program introduces a new approach of food education that includes experiential learning activities, assisting children in developing autonomous food choice skills and improving their nutritional literacy [109].

The success of these initiatives is inextricably linked to the institutionalized cooperation among the Ministries of Health, Education, and Agriculture, evidenced by data from the School Meals Coalition, which shows that Finland has more than 95% coverage of the student population receiving daily school meals [110].

### 5.2. European Framework for Child Nutrition and “Hidden Hunger”

At the European level, addressing malnutrition and “hidden hunger” remains a high policy concern. The European Commission has allocated an amount exceeding EUR 3.4 billion between 2020 and 2030 to boost micronutrient access, notably among vulnerable child populations [111].

At the same time, the Horizon Europe program finances research projects that explore the degrees of micronutrient deficiencies across Europe, underlining the necessity for fortification and vitamin-enriched meals in school lunch programs [112].

Furthermore, organizations such as the World Food Programme and Concern Worldwide are conducting EU-funded projects to combat child undernutrition through meal distribution, educational activities, and health indicator monitoring [113].

The link between scientific evidence and policymaking is important to the long-term viability of these initiatives. Epigenetic research has found that early-life nutritional exposures, particularly throughout childhood, influence gene regulation related with metabolism and the risk of chronic disease [114].

Verhagen (2019) describes how incorporating such findings into public health policies has resulted in interventions aimed at important life stages, from maternal nutrition to school age [115]. However, Dupras et al. (2019) emphasize the ethical and legal issues that arise when translating epigenetic research into policy actions [116], whilst recent critiques, such as those published by *Practical Ethics* (2023), highlight the need for transparency and scientific accountability [117].

Overall, the Finnish experience and European initiatives demonstrate how evidence-based policy, founded on scientific research, may significantly enhance the nutritional health of children aged 5 to12.

## 6. Socioeconomic Inequalities and Dietary Quality

Socioeconomic disparity has a major influence on children’s nutrition. According to Darmon and Drewnowski (2008), socioeconomic class is directly related to diet quality, as low-income households have less access to fresh and nutrient-dense meals and are more likely for choose cheaper, energy-dense options [118]. Consuming such foods leads to a “double burden” nutritional deficiency and an increased risk of juvenile obesity [119].

According to The World Health Organization’s research on inequalities in child nutrition in Europe, children from low-income households are 40% less likely to consume fruits and vegetables on a daily basis, and they possess much higher rates of anemia and vitamin D deficiency [120]. Similarly, the Health Behaviour in School-aged Children (HBSC) study found that socioeconomic level is positively correlated with healthy eating behaviors, such as regular breakfast consumption and reduced soft drink intake [121].

Comparable evidence appears from Spain, where Pérez-Farinós et al. (2013) discovered that wealth gaps are reflected in children’s diet quality, with lower consumption of fruits, fish, and dairy products, but higher intake of sweets and fried meals [119]. These findings indicate that malnutrition in affluent cultures is not limited to food deprivation, but also manifests as qualitative undernutrition: insufficient nutrient intake caused by excessive consumption of ultra-processed foods.

## 7. The Commercial Pressure of the Food Industry

Aside from social inequalities, children’s diets are impacted by the commercial influence of the food industry. Nestle (2018) points out that food businesses frequently falsify scientific findings to portray their goods as healthy, which contributes to consumer ignorance and incorrect perceptions of nutritional values [122].

Pursuant to the WHO’s study on commercial determinants of health, advertising of unhealthy items targets school-aged children systematically via television, online platforms, and digital games [123]. These tactics boost children’s predilection for foods heavy in sugar, salt, and saturated fat, while decreasing their consumption of healthful foods.

Harris et al. (2009) outline in detail the “food marketing defense model,” which explains how repeated exposure encourages children to form emotional attachments to commercial brands, leading them to choose harmful items against parental warnings [124]. Similarly, Moodie et al. (2013), suggest, in their significant *Lancet* publication, that the promotion of ultra-processed foods is carried out using strategies similar to those employed by the tobacco industry, such as political lobbying, aggressive marketing, and deceptive scientific communication [125].

This persistent commercial push exacerbates preexisting social inequities. Children from lower-income families, who are more exposed to television and digital information, are the primary target audience for such commercials [123]. Thus, nutritional inequality persists not only via economic access to food, but also through cultural structuring of dietary norms.

## 8. Nutrition During School Age: A Critical Developmental Window

The school years (ages 5 to 12) are critical for developing food habits, physical growth, and long-term health effects. Food marketing through social media and other media channels plays a significant role in shaping children’s dietary preferences and consumption patterns. Digital advertising, influencer content, and in-store promotions predominantly market energy-dense, nutrient-poor foods, while young children remain particularly vulnerable due to limited ability to recognize persuasive intent. This pervasive marketing environment can undermine nutrition education efforts, underscoring the need for stronger regulation and media literacy interventions to protect children’s dietary health. During this stage, nutrition influences not only physical growth, but also cognitive ability, focus, and academic accomplishment [126].

Diet quality during school years is directly related to the prevention of childhood obesity, which is still a serious public health concern across Europe [127,128,129]. Insufficient intake of fruits, vegetables, and whole grains, combined with the overconsumption of ultra-processed foods, has been linked to metabolic disorders and higher body mass index [130]. As result, investing in policies that foster healthy eating environments in schools is critical for improving health at a young age.

## 9. European Policy Context: Toward a Coordinated Approach

At the European level, a cohesive and coordinated policy response is critical. The EU School Scheme, which encourages the distribution of fruits, vegetables, and milk in schools, is one of the few collaborative programs that connect nutrition policy with education and agriculture [131]. However, its execution varies among Member States, with variations in product frequency, food, quality, and nutritional standards [132].

Both the WHO [123] and the FAO [132] emphasize that the success of such programs depends on a robust institutional framework, intersectoral cooperation, and long-term funding.

The SchoolFood4Change strategy [133] further emphasizes the need for policies that link school nutrition to sustainability and local food systems, encouraging both food education and student participation in meal selection. Similarly, the FAO and World Food Programme (WFP) [134] suggest an Integrated School Food and Nutrition Framework that includes healthy food distribution, health education, and secure meal access infrastructure.

## 10. Protecting Children from Harmful Food Marketing

The call to action goes beyond governments and includes all key parties. The WHO Guideline on Policies to Protect Children from the Harmful Impact of Food Marketing underlines the importance of policymakers implementing enforceable measures to prohibit the commercial promotion of unhealthy foods to children [128].

At the same time, health professionals and educators play key roles in promoting positive dietary patterns through nutrition education and the enhancement of food literacy within schools [135]. Collaboration among these actors is vital for the success of interventions.

Meta-analyses indicate that school-based interventions combine teaching programs, changes in canteen options, and parental engagement, significantly improving fruit and vegetable intake while decreasing sugar consumption [136,137]. As a result, adopting comprehensive, evidence-based programs is the most effective way to promote healthy eating behaviors among primary school students.

## 11. Conclusions and Final Review

Childhood obesity and undernutrition are two sides of the same coin, imposing a double burden on public health systems across Europe [1,2,50]. Despite numerous policy frameworks, such as the EU Action Plan on Childhood Obesity (2014–2020) and the WHO European Food and Nutrition Action Plan (2015–2020), progress has been uneven, and prevalence rates of childhood overweight and obesity rates continue to rise. The lack of harmonized data collection, inconsistent implementation of national policies, and poor enforcement of food labelling and marketing regulations remain major barriers to effective prevention [8,128].

Significant geographical inequalities persist. Northern European countries, such as Sweden and Denmark, report the lowest obesity rates, whereas Southern European countries, including Greece, Italy, and Spain, face some of the highest [50]. These disparities reflect broader socioeconomic and cultural determinants, as children from disadvantaged backgrounds are more likely to experience both undernutrition and obesity due to limited access to nutritious foods and health services [13]. Migrant populations, in particular, face elevated vulnerability linked to acculturation stress, barriers in healthcare access, and food insecurity [15]. This coexistence of undernutrition and overnutrition within the same populations exemplifies the double burden of malnutrition, posing complex challenges for health systems.

Beyond socioeconomic disparities, biological and developmental factors play crucial roles. Nutrition during the first 1000 days of life, from conception to two years of age, is critical for long-term health outcomes. Maternal malnutrition, both under- and overnutrition, has lasting implications for fetal growth, metabolic programming, and disease susceptibility [26,28]. Breastfeeding, appropriate complementary feeding, and balanced infant nutrition have been consistently associated with reduced risks of obesity, improved immunity, and healthy neurodevelopment [37,39]. Epigenetic mechanisms further underline how early-life nutritional exposures can influence gene expression and disease risk across generations [22,30].

To effectively address childhood malnutrition in all its forms, Europe must adopt a holistic, multisectoral approach. Early nutrition education should not be viewed as an isolated health action, but as a long-term investment in population well-being. Integrating nutrition literacy into school curricula, providing universal access to healthy school meals, and promoting sustainable food environments can help shape lifelong dietary habits [1,41]. Fiscal policies, such as taxing sugar-sweetened beverages and subsidizing fruits and vegetables, have shown potential to make healthy diets more accessible [12].

A unified European framework is now required to combine early nutrition, education, and social protection. Establishing a unified EU-wide monitoring system for child nutrition, strengthening political commitment, and supporting continuous research on early nutrition, microbiome, and epigenetic factors will be key to reducing inequalities and improving outcomes [6,22]. Policymakers should give structural and financial support, health professionals must provide evidence-based guidance, and educators should promote nutritional literacy as part of everyday learning.

Only by implementing this integrated and coordinated plan will Europe ensure that all children, regardless of socioeconomic status, grow up well-nourished, resilient, and capable of reaching their full potential. A concerted strategy that links health, education, and sustainability will not only reduce childhood obesity, but will also lay the groundwork for future generations to be healthier and more equal.

## Data Availability

No new data were created or analyzed in this study.

## Abbreviations

The following abbreviations are used in this manuscript:

WHO	World Health Organization
BMI	Body Mass Index
COSI	Childhood Obesity Surveillance Initiative
CRP	C-Reactive Protein
DOHaD	Developmental Origins of Health and Disease
IGF-1	Insulin-like Growth Factor-1
EU	European Union
SAFE	Safe Food Advocacy Europe
EPHA	European Public Health Alliance
NCDs	Noncommunicable Diseases
SBB	Sugar-Sweetened Beverages
FBDGs	Food-Based Dietary Guidelines
EFSA	European Food and Safety Authority
JRC	Joint Research Center
MUST	Malnutrion Universal Screening Tool
FAO	Food and Agriculture Organization
HBSC	Health Behaviour in School-Aged Children
WFP	World Food Programme
STAMP	Screening Tool for the Assessment of Malnutrition in Pediatrics
